# Helmert Variance Component Estimation for Multi-GNSS Relative Positioning

**DOI:** 10.3390/s20030669

**Published:** 2020-01-25

**Authors:** Mowen Li, Wenfeng Nie, Tianhe Xu, Adria Rovira-Garcia, Zhenlong Fang, Guochang Xu

**Affiliations:** 1 Institute of Space Sciences, Shandong University, 180 Wenhuaxi Road, Weihai 264209, China; mowenli@mail.sdu.edu.cn (M.L.); wenfengnie@sdu.edu.cn (W.N.); zhlfang@foxmail.com (Z.F.); xuguochang@hit.edu.cn (G.X.); 2 Shandong Provincial Key Laboratory of Optical Astronomy and Solar-Terrestrial Environment, Shandong University, Weihai 264209, China; 3 State Key Laboratory of Geo-Information Engineering, Xi’an Research Institute of Surveying and Mapping, Xi’an 710054, China; 4 Research group of Astronomy and Geomatics (gAGE), Universitat Politecnica de Catalunya (UPC), 08034 Barcelona, Spain; 5 Institute of Space Science and Applied Technology, Harbin Institute of Technology, Shenzhen 518000, China

**Keywords:** Multi-GNSS, Helmert variance component estimation (HVCE), weighting strategy, relative positioning

## Abstract

The Multi-constellation Global Navigation Satellite System (Multi-GNSS) has become the standard implementation of high accuracy positioning and navigation applications. It is well known that the noise of code and phase measurements depend on GNSS constellation. Then, Helmert variance component estimation (HVCE) is usually used to adjust the contributions of different GNSS constellations by determining their individual variances of unit weight. However, HVCE requires a heavy computation load. In this study, the HVCE posterior weighting was employed to carry out a kinematic relative Multi-GNSS positioning experiment with six short-baselines from day of year (DoY) 171 to 200 in 2019. As a result, the HVCE posterior weighting strategy improved Multi-GNSS positioning accuracy by 20.5%, 15.7% and 13.2% in east-north-up (ENU) components, compared to an elevation-dependent (ED) priori weighting strategy. We observed that the weight proportion of both code and phase observations for each GNSS constellation were consistent during the entire 30 days, which indicates that the weight proportions of both code and phase observations are stable over a long period of time. It was also found that the quality of a phase observation is almost equivalent in each baseline and GNSS constellation, whereas that of a code observation is different. In order to reduce the time consumption of the HVCE method without sacrificing positioning accuracy, the stable variances of unit weights of both phase and code observations obtained over 30 days were averaged and then frozen as a priori information in the positioning experiment. The result demonstrated similar ENU improvements of 20.0%, 14.1% and 11.1% with respect to the ED method but saving 88% of the computation time of the HCVE strategy. Our study concludes with the observations that the frozen variances of unit weight (FVUW) could be applied to the positioning experiment for the next 30 days, that is, from DoY 201 to 230 in 2019, improving the positioning ENU accuracy of the ED method by 18.1%, 13.2% and 10.6%, indicating the effectiveness of the FVUW.

## 1. Introduction

Since the United Stated Global Positioning System (GPS) became fully operational in 1995 (https://www.gps.gov), the Global Navigation Satellite System (GNSS) continuously developed toward a Multi-constellation GNSS (Multi-GNSS) paradigm [1]. The Russian GLObal NAvigation Satellite System (GLONASS) is adopting a code division multiple access (CDMA) signal, substituting the original frequency division multiple access (FDMA) (https://www.glonass-iac.ru/). The European Galileo plans to complete Galileo constellation with 30 satellites by 2020 (http://www.esa.int/Applications/Navigation/Galileo/What_is_Galileo). For the BeiDou Navigation Satellite System (BDS) of China, BDS-2 and BDS-3 satellites are currently used together (http://www.beidou.gov.cn/), and BDS-3 will be completed by 2020 for a global service [2].

By providing more satellites and signals, Multi-GNSS can benefit the GNSS community in many aspects. It can not only improve the positioning accuracy for both precise point positioning (PPP) and real-time kinematics (RTK), but also shorten convergence time to obtain the final positioning accuracy more quickly [3,4,5,6]. In the Multi-GNSS context, the International GNSS Service (IGS) initialized the Multi-GNSS EXperiment (MGEX) in 2014 [7], aiming to provide Multi-GNSS products such as precise orbit, clock and satellite bias. Since then, MGEX products have enabled Multi-GNSS applications, such as global ionosphere modelling [8], water vapor determination [9], precise agriculture [10], crustal deformation [11] and GNSS-Reflectometry-based altimetry [12].

It is well known that the quality of the measurements from different GNSS constellations is not homogenous. Among other reasons, the discrepancies can be attributed to the following: the satellite orbits are different not only between GNSSs but also within the same constellation. For instance, BDS contains three types of orbit satellites, medium Earth orbit (MEO), geostationary Earth orbit (GEO) and inclined geo-synchronous orbit (IGSO) [13,14]; the signal structure adopted by GPS, BDS and Galileo is CDMA, while that by GLONASS is FDMA.

Therefore, many weighting methods are proposed in the literature to determine the weight of GNSS according to the quality of observations: the carrier-to-noise ratio [15], elevation-dependent (ED) weighting method [16], azimuth-dependent-elevation weighting model [17] and the method based on signal-in-space ranging errors (SISRE) information [18], among others. Variance component estimation (VCE) can also be used to adjust the contribution of different GNSS constellations by determining their individual variances of unit weight [19]. The theoretical algorithm of the Helmert variance component estimation (HVCE) was deeply studied [20,21,22] and a simplified version of the rigorous HVCE was proposed [23].

From then on, HVCE was applied to many different geodetic areas [24,25,26,27]. For positioning applications, the algorithm of HVCE was simplified in Kalman filtering to save computation load and to achieve good convergence time in GPS single point positioning (SPP) [23]. Robust HVCE can provide suitable weights for various observation groups and guarantee the reliability of the positioning results in network adjustment [28]. As for Multi-GNSS applications, HVCE was proven to perform well in Multi-GNSS time and frequency transfer [29], the Inertial Navigation System (INS) tightly coupled integrated with Multi-GNSS PPP [30] and Multi-GNSS positioning. HVCE can determine the weight matrix of GPS/BDS observations, reaching horizontal accuracy of 0.2 m in pseudorange differential positioning [31]. The modified VCE and HVCE were combined in GPS/BDS PPP, which significantly improved the positioning accuracy and reduced the convergence time [32]. Finally, HVCE applied in GPS/BDS/GLONASS pseudorange-based relative positioning improved positioning accuracy by 11.5% [33].

However, these aforementioned works paid more attention to the improvement in positioning accuracy obtained by HVCE posterior weighting method, rather than to the time-varying features of the variances of unit weight and their further applications. Therefore, our present study focuses on the long-term time-varying characteristic of HVCE weights proportion of Multi-GNSS, by analyzing variances of unit weights of both phase and code observations. Based on the one-month stability of HVCE weight proportions, variances of unit weight were applied as prior information for the test of the next month, which was found to be efficient at enhancing positioning accuracy and time-saving in the Multi-GNSS process.

The paper is organized as follows. We first present the theory of HVCE robust Kalman filtering and its algorithm implementation in Section 2. Then, Section 3 introduces the experimental setups and station status during the experimental data campaign. The results of accuracy improvements achieved by HVCE posterior weighting-based Multi-GNSS positioning are presented in Section 4. Finally, we discuss experimental results associating with previous works and summarize all significant conclusions in Section 5.

## 2. Method

### 2.1. Helmert Variance Component Estimation for Robust Kalman Filtering

Different types of orbit, signal structure, data quality and measurement type, require different GNSS observations to be properly weighted. The HVCE-based robust Kalman filtering can balance the contributions of different grouped data and provide individual variances of unit weights. In what follows, we provide the rigorous deduction of HVCE-based robust Kalman filtering for Multi-GNSS positioning.

The classic Kalman filtering solution Equations can be expressed as

(1) $$\hat{X}\;=\;\bar{X}\;+\;K(L\;-\;A\bar{X})$$

(2) $${\hat{Q}}_{X}\;=\;(I\;-\;KA){\bar{Q}}_{X}$$

(3) $$K\;=\;{\bar{Q}}_{X}A^{T}{\left(A{\bar{Q}}_{X}A^{T}\;+\;Q_{V}\right)}^{-1},$$

where $\bar{X}$ and ${\bar{Q}}_{X}$ are the predicted state parameters and its covariance matrix; $\hat{X}$ and ${\hat{Q}}_{X}$ are their estimated values, respectively; L and $Q_{V}$ are an observation vector and its covariance matrix; A is coefficient matrix of predicted state parameters; K is called gain matrix; and I denotes the unit matrix. One popular prior weighting strategy used in classic Kalman filtering to calculate observation covariance matrix is the ED weighting method:

(4) $$Q_{Vi}=\;k_{ratio}\sin^{-2}\theta_{i}\cdot \varepsilon^{2}{}_{phase}$$

where the $Q_{vi}$ and $\theta_{i}$ are the variance and the satellite elevation angle of the i th observation; $\varepsilon^{2}{}_{phase}$ is the given variance of phase observation with $\varepsilon_{phase}$ set as 3 mm in this paper; and $k_{ratio}$ denotes a noise-ratio of $k_{ratio}\;=\;1$ for phase observations, while $k_{ratio}\;=\;100^{2}$ for code observations.

However, the classic Kalman filtering is easily affected by the outlier in observations, and in our study, the observations are dependent after being double-differenced to implement relative positioning. Therefore, the weight matrix of L has to be modified as [34]:

(5) $${\left({\bar{P}}_{V}\right)}_{ij}\;=\;\sqrt{\gamma_{ii}\gamma_{jj}}{\left(P_{V}\right)}_{ij}$$

(6) $$\gamma_{ii}\;=\;\{\begin{array}{c} 1\\ \frac{c}{R_{i}/\sqrt{{\left(Q_{V}\right)}_{ii}}} \end{array}\begin{array}{c} \left|R_{i}/\sqrt{{\left(Q_{V}\right)}_{ii}}\right|\;\leq \;c\\ \left|R_{i}/\sqrt{{\left(Q_{V}\right)}_{ii}}\right|\;>\;c \end{array}\;$$

(7) $$P_{V}\;=\;Q_{v}^{-1}$$

where subscript i and j are the i th row and j th column of a specified matrix; $\gamma_{ii}$ and $\gamma_{jj}$ are two reduction factors; $R_{i}$ is the residual of the $L_{i}$; c denotes a constant threshold that is usually chosen as 1.3–2.0.

This yields a robust Kalman filtering with the updated observation covariance matrix ${\bar{Q}}_{v}$:

(8) $$K\;=\;{\bar{Q}}_{X}A^{T}{\left(A{\bar{Q}}_{X}A^{T}\;+\;{\bar{Q}}_{V}\right)}^{-1}$$

(9) $${\bar{Q}}_{V}\;=\;{\bar{P}}_{V}^{-1}.$$

Let us assume that there are m groups of data according to code and phase observations of different GNSSs at a given epoch. Associating Equations (2) and (3) with HVCE theory [19,20,21,22], the solution of variance of unit weight of each individual observation group can be expressed as

(10) $$\left[\begin{array}{c} \sigma_{1}^{2}\\ \sigma_{2}^{2}\\ \vdots \\ \sigma_{m}^{2} \end{array}\right]\;=\;{\left[\begin{array}{cccc} s_{1,1} & s_{1,2} & \cdots & s_{1,m}\\ s_{2,1} & s_{2,2} & \cdots & s_{2,m}\\ \vdots & \vdots & \ddots & \vdots \\ s_{m,1} & s_{m,2} & \cdots & s_{m,m} \end{array}\right]}^{-1}\;\left[\begin{array}{c} R_{1}^{T}{\bar{Q}}_{V1}^{-1}R_{1}\\ R_{2}^{T}{\bar{Q}}_{V2}^{-1}R_{2}\\ \vdots \\ R_{m}^{T}{\bar{Q}}_{Vm}^{-1}R_{m} \end{array}\right]$$

(11) $$\begin{array}{r} s_{i,i}\;=\;n_{i}\;-\;2tr(N_{i}N^{-1})\;+\;tr(N_{i}N^{-1}N_{i}N^{-1})\\ s_{i,j}\;=\;s_{j,i}\;=\;tr(N_{i}N^{-1}N_{j}N^{-1})\\ N_{i}\;=\;A_{i}^{T}Q_{Vi}^{-1}A_{i}\\ N^{-1}\;=\;{\hat{Q}}_{X} \end{array}\},$$

where $\sigma_{i}^{2}$ is the estimated variance of unit weight and ‘tr’ stands for trace of a matrix. $R_{i}$, $N_{i}$ and $n_{i}$ stand for the residual vector, normal matrix and observation number of the ith group, respectively. According to the variance of unit weight solution calculated from Equations (10) and (11), one can conveniently update the covariance matrix of the i th observations group using:

(12) $${\tilde{Q}}_{Vi}\;=\;\frac{\sigma_{i}^{2}}{c_{0}}Q_{Vi},i\;=\;1,2,\ldots ,m$$

(13) $$K\;=\;{\bar{Q}}_{X}A^{T}{\left(A{\bar{Q}}_{X}A^{T}\;+\;{\tilde{Q}}_{V}\right)}^{-1},$$

where ${\tilde{Q}}_{Vi}$ represents the updated covariance matrix of the i th observations group.

It should be noted that $c_{0}$ is an arbitrary constant, which is usually one of the estimated variances of a unit weight. Considering the GNSS consistency, we chose $\sigma_{G,L}^{2}$ of GPS phase observations group as the substitute for $c_{0}$. Once the observation covariance matrix is updated, Equation (3) can be replaced by Equation (13) and used to calculate new state parameters.

### 2.2. Flow Chart of Multi-GNSS HVCE for Robust Kalman Filtering Algorithm

**Figure 1:**
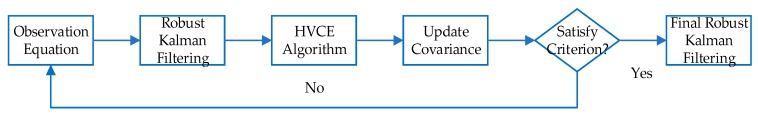
Flow chart of Multi-GNSS HVCE for robust Kalman filtering.

In the present paper, an iterative algorithm is presented to calculate the final solution of variance of unit weight $\sigma^{2}$ and state parameters. The flow chart of the algorithm is presented in Figure 1. At the start of the loop, Equations (1)–(9) are used to calculate an initial $\hat{X}$ and residual vector R of robust Kalman filtering. Then, the variance of unit weight $\sigma^{2}$ of phase and code observations of GNSSs are calculated using HVCE algorithm expressed by Equations (10) and (11). At the end of the iteration, each observation group’s covariance matrix ${\tilde{Q}}_{Vi}$ is updated using Equation (12) and combined to a new covariance matrix ${\tilde{Q}}_{V}$ for the robust Kalman filtering of next iteration.

The end of the loop depends on the availability of variance of unit weight $\sigma^{2}$ of each observation group. According to HVCE theory, the final solution should satisfy the criterion that all variances of unit weight $\sigma^{2}$ of different observation groups are numerically equal. However, in the real implementation of the iterative algorithm, the obtainable result is a cluster of approximately equal variances of unit weight $\sigma_{i}^{2}$. Assuming that the criterion of final solution is satisfied after several iterations, it is deduced the equivalent critical Equation for all observation groups as

(14) $$\left|\frac{\sigma_{i,z}^{2}}{c_{0,z}}\;-\;1\right|\;\leq \;\delta ,\;c_{0,z}\;=\;\sigma_{G,L}^{2},$$

where z is the number of the iterations. δ denotes the threshold value of criterion, which is a diminutive value close to zero (δ  =  0.01 is used in our experimentation). Therefore, the final solution of variance of unit weight of the i th observation group with respect to the original covariance matrix can be expressed as

(15) $$\sigma_{i}^{2}\;=\;\prod_{j\;=\;1}^{z}\frac{\sigma_{i,j}^{2}}{c_{0,j}},$$

where the final variances of unit weight $\sigma_{i}^{2}$ of phase and code are employed as the representations of weight proportion for the further analysis in our study.

Before the experiment, we set the algorithm to iterate 7 times (z  =  7) to calculate final solution for variances of unit weights of observation groups. As a result, the variances of unit weight reached 97.2%, 99.5% and 99.9% of the final solution after the first, second and third iteration on average, which means that most of loops will stop after the second iteration, as the δ is set as 0.01.

## 3. Experiment Setup

### 3.1. Station Selection

**Figure 2:**
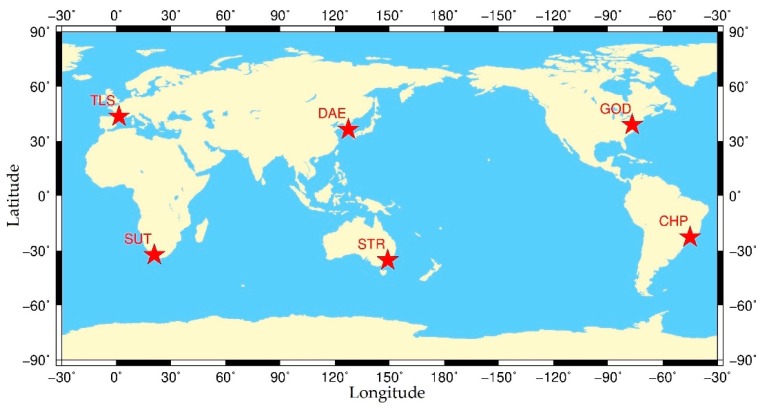
Locations of six independent baselines with co-located International GNSS Service (IGS) stations.

For the purpose of evaluating the availability of HVCE posterior weighting strategy in robust Kalman filtering and analyzing the weight proportions of GPS, BDS, GLONASS and Galileo in different areas, we selected twelve IGS stations located in five different continents to form six independent, short baselines shown in Figure 2. The basic information of all stations and baseline distributions for relative positioning is shown in Table 1.

Relative positioning with short baselines was selected as the experimental strategy because it eliminates satellite and receiver-dependent errors (e.g., clock error and hardware delays) and most parts of signal propagation-dependent errors (e.g., ionosphere and troposphere delays) [35].Therefore, the unknown parameters estimated in the HVCE-based robust Kalman filtering are the coordinates of rover station, the ambiguities and the inter-frequency bias (IFB) of GLONASS observations when the baseline receivers are of different types.

### 3.2. Data Processing Strategy

Our daily kinematic relative positioning uses: single-frequency pseudo-range and carrier phase observations, i.e., GPS L1, BDS B1, GLONASS L1 and Galileo E1, MGEX broadcast ephemeris, and both BDS-2 and BDS-3 satellites, including MEO, GEO and IGSO. The reason that only single-frequency observations are used is to avoid the influence of the second frequency observations in the determination of variance of unit weight for each constellation. The campaign dated from 2019 and it is divided into two parts of one month each, from day of year (DoY) 171 to 200 and from DoY 201 to 230, detailed in what follows. The initial noise levels of the pseudorange and carrier-phase of all GNSS constellations are set as 0.3 m and 0.003 m, respectively. Besides, a white noise of 30 m is applied to each component of the coordinate parameters in the Kalman filtering. Considering the computational efficiency, the data sampling interval is set to 180 s. The positioning experiments are computed by a Red Hat Enterprise Linux Server with the 1.60 GHz CPU.

For the purpose of evaluating the weight proportion of Multi-GNSS in different baselines, five strategies are proposed to carry out the kinematic relative positioning experiment: ED prior weighting-based GPS-only and Multi-GNSS (G + C + R + E) strategy (ED GPS-only and ED Multi-GNSS for short), and their corresponding HVCE posterior weighting-based strategies (HVCE GPS-only and HVCE Multi-GNSS for short). Finally, in order to obtain the high accuracy result and reduce the time consumption of the HVCE method at the same time, the variances of unit weight over the 30 days are averaged and then frozen as a priori information in the positioning experiment, named frozen variances of unit weight (FVUW). The Multi-GNSS FVUW strategy is extended to the positioning experiment for 30 days from DoY 201 to 230.

Note that the observations from 00:00:00 DoY 192 to 12:15:46 DoY 193 for station GODN and observations of DoY 171 for station TLSG in Receiver INdependent EXchange (RINEX) format are missing in the IGS archive (ftp://cddis.gsfc.nasa.gov/pub/gps/data/daily/). The final coordinate solution of DoY 185 provided by IGS is used as the reference to calculate root mean square (RMS), and the station coordinate (x, y, z) components in the WGS84 reference frame are transformed into ENU components. The RMS values are computed from the epoch when the 3D positioning error is continually lower than 0.1 m. Particularly, the averaged convergence time and number of available positions whose 3D positioning errors are below 0.1 m are shown in Table 2.

Before assessing the experimental results, the average number of common satellites and positioning dilution of precision (PDOP) values of individual and combined GNSSs in each baseline during the experiment are presented in Table 3.

It can be read that Multi-GNSS (G + C + R + E) provides the larger number of available satellites for all baselines. As a result, the PDOP values of Multi-GNSS are smaller than single-GNSS. Focusing on the performance of different GNSSs, GPS and GLONASS provide more consistent positioning conditions, with average numbers of 7.7 and 5.9 satellites and corresponding PDOP values of 1.2 and 1.5 for all baselines. For Galileo, the average number of available satellites is stable at approximately 5.5 with the PDOP value of 1.7 in the most of baselines except DAE in Asia. In the case of BDS, as the BDS-3 constellation is under construction and BDS-2 constellation has been completed, the performance of BDS is much better than any other systems in Asia-Pacific (DAE, STR), with more than 10 available satellites, and its PDOP value approaches to 1. In this regard, BDS can provide equally good satellite geometry as GPS and GLONASS do in South Africa (SUT). However, BDS has a limited advantage in America (CHP, GOD) and Europe (TLS) with less than six available satellites.

## 4. Experimental Results and Discussion

In the present section, both phase and code observation weight proportions of all Multi-GNSS baselines are discussed separately at first. Afterwards, the positioning performance of HVCE posterior weighting method is evaluated for all baselines. Finally, based on the stability of the weight proportion of both phase and code observations, the effectiveness of FVUW Multi-GNSS strategy in positioning is assessed.

### 4.1. Weight Proportions of Multi-GNSS Phase and Code Observations

**Figure 3:**
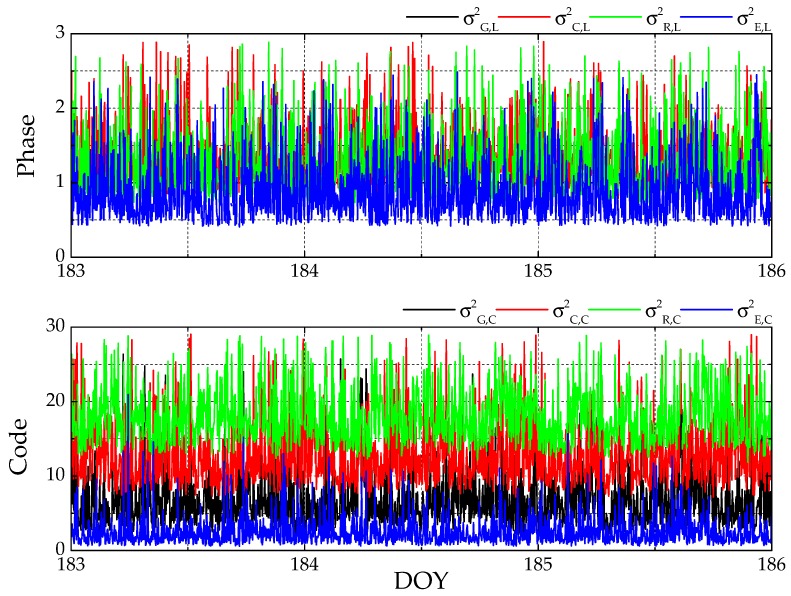
Time series of variances of unit weight of phase (**top**) and code (**bottom**) for baseline South Africa (SUT) from day of year (DoY) 183 to 186 in 2019.

As mentioned in Section 2, the variance of unit weight generated from the HVCE posterior weighting strategy is a criterion to evaluate the quality of different observation types from various GNSSs. The variances of unit weight in a time series of baseline SUT obtained by HVCE Multi-GNSS are shown as an example in Figure 3, where the variances of unit weights of both phase and code jump around a certain value are indicated from epoch to epoch, and the relationship among the variances of unit weight of different GNSSs is stable within a day. Hence in this section, the daily average variances of unit weight obtained by HVCE Multi-GNSS strategy are analyzed in detail to assess the weight proportion of phase and code separately.

**Figure 4:**
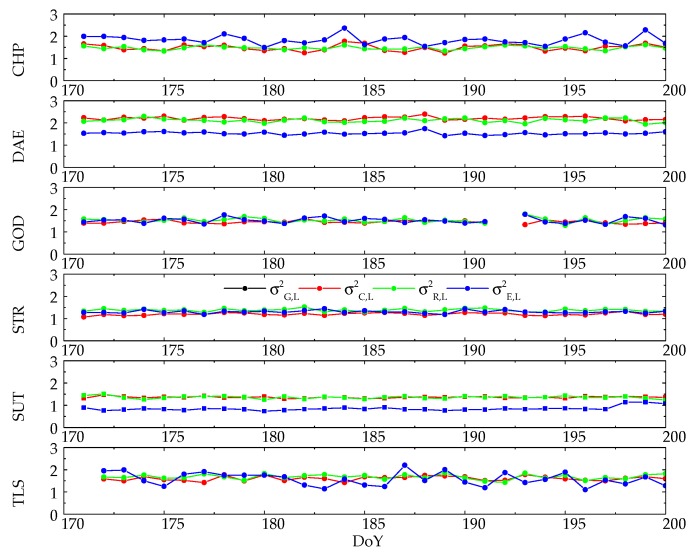
Daily average variance of unit weight of Multi-GNSS phase observations in different baseline tests.

Since phase observations play a more significant role in achieving high accuracy positioning, the weight proportion of phase observations has to be treated carefully. Figure 4 presents the time series of daily averaged phase variance of unit weight for the whole 30 days. Note that the GPS phase variances of unit weight $\sigma_{G,L}^{2}$ are not shown because its value is 1.0, by definition in Equation (12).

It can be seen that all phase variances of unit weights of BDS, GLONASS and Galileo are higher than 1.0 and lower than 2.5 with a rather similar pattern. However, the result differs from baseline to baseline. The phase weight proportions in baselines DAE, GOD, STR and SUT present a more stable feature than the other two baselines. In baselines DAE and SUT, the variances of unit weight $\sigma_{C,L}^{2}$ and $\sigma_{R,L}^{2}$ are close to each other and higher than $\sigma_{E,L}^{2}$, while the difference between $\sigma_{C,L}^{2}$, $\sigma_{R,L}^{2}$ and $\sigma_{E,L}^{2}$ is not obvious in baselines GOD and STR. For baselines CHP and TLS, the phase weight proportion fluctuates over time, and $\sigma_{E,L}^{2}$ is slightly higher than $\sigma_{C,L}^{2}$ and $\sigma_{R,L}^{2}$ in CHP, while the phase variances of unit weight are similar in TLS.

**Figure 5:**
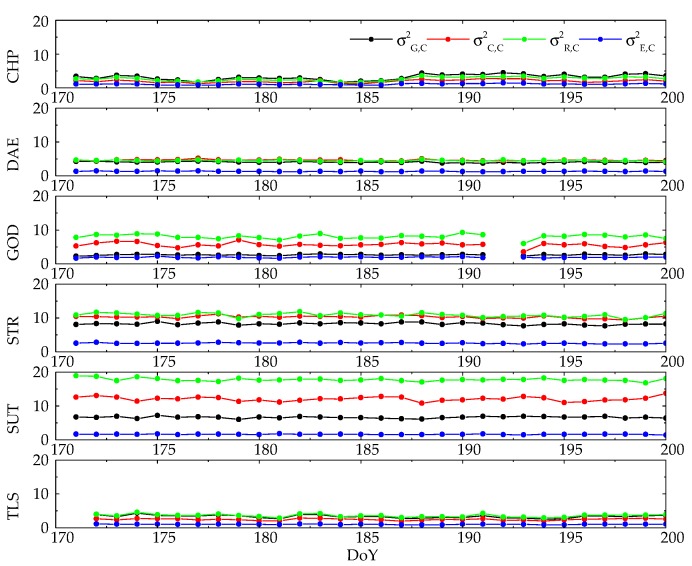
Daily average variance of unit weight of Multi-GNSS code observations in different baseline tests.

On the contrary, the discrepancy of code weight proportion between different baselines is quite evident. In this part, the GPS code unit weight $\sigma_{G,C}^{2}$ is estimated because the GPS phase unit weight $\sigma_{G,L}^{2}$ is set as the reference. It should be noted that the initial noise ratio between GPS phase and code is $k_{ratio}\;=\;100^{2}$, as mentioned in Section 2. Figure 5 depicts differences of code variance of unit weight for GPS, BDS, GLONASS and Galileo are obvious in all baselines. It is found that $\sigma_{R,C}^{2}\;>\;\sigma_{C,C}^{2}\;>\;\sigma_{G,C}^{2}\;>\;\sigma_{E,C}^{2}$ in the test of GOD, STR and SUT, and the code variances of unit weight ranging from 2.5 to 11.0 in baseline GOD and STR, while $\sigma_{R,C}^{2}$ reaches 17.8 in SUT. In baseline DAE, the $\sigma_{G,C}^{2}$, $\sigma_{C,C}^{2}$ and $\sigma_{R,C}^{2}$ are similar to each other, while the $\sigma_{E,C}^{2}$ is the smallest one. The relationships $\sigma_{G,C}^{2}\;>\;\sigma_{R,C}^{2}\;>\;\sigma_{G,C}^{2}\;>\;\sigma_{E,C}^{2}$ and $\sigma_{R,C}^{2}\;>\;\sigma_{R,C}^{2}\;>\;\sigma_{C,C}^{2}\;>\;\sigma_{E,C}^{2}$ are found in CHP and TLS.

As the variances of unit weight for both phase and code observations present such consistency from day to day, we averaged their values for the entire 30 days period for all baselines; see Table 4. The RMS values of all variances of unit weight over 30 days for all baselines are also shown as their superscripts in Table 4, which is 0.3–0.5 of the corresponding $\sigma^{2}$ value. From the last row of Table 4, it is confirmed that the weight proportion of phase is almost equal among GPS, BDS, GLONASS and Galileo, as their average variances of unit weight are nearly 1.0 in all baselines. Generally, the variances of unit weight of $\sigma_{C,L}^{2}$, $\sigma_{R,L}^{2}$ and $\sigma_{E,L}^{2}$ are higher than 1.4 in baseline CHP, DAE, GOD and TLS, which means that the phase observations of GPS present a smaller variance than other three GNSSs in these baselines. On the other hand, phase observations from different GNSSs express a more coincident variance in baseline STR and SUT, as their phase variances of unit weight are closer to 1.0.

From the right panel in Table 4, the average code variances of unit weight present a higher variability than phase variances of unit weight. From the view of GNSS constellations, the average code variance of unit weight of Galileo $\sigma_{E,C}^{2}$ is 1.59, which is the lowest value in comparison with the other three GNSSs and indicates the outstanding performance of Galileo code observations. However, the relationship of the code variances of unit weights among GPS, BDS and GLONASS differs between baselines, as discussed in the previous paragraph, and the average values of these three GNSSs are higher than 4.5 with the sequence of $\sigma_{R,C}^{2}\;>\;\sigma_{C,C}^{2}\;>\;\sigma_{G,C}^{2}$.

### 4.2. Accuracy of HVCE Posterior Weighting-Based Multi-GNSS Positioning

**Figure 6:**
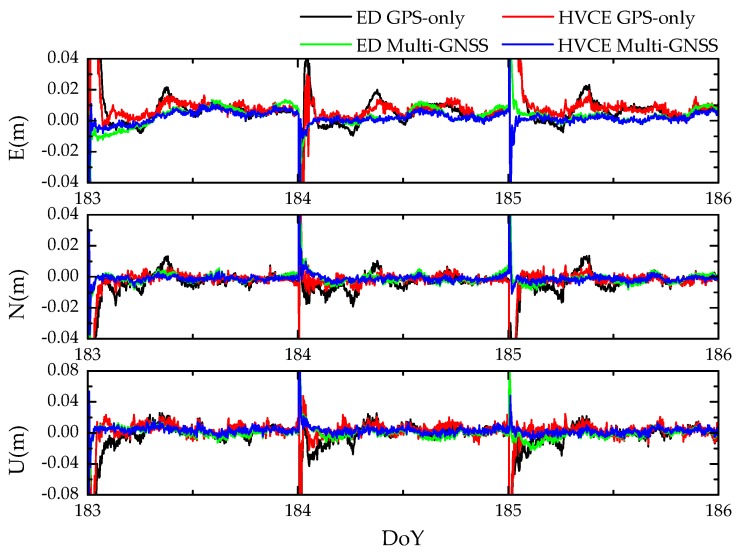
Time series of kinematic positioning errors of east (**top**), north (**middle**) and up (**bottom**) components for baseline SUT from DoY 183 to 186 in 2019. The reset appearing in the start of each day is caused by the independent daily process.

The positioning accuracy is a critical factor to evaluating the performance of HVCE Multi-GNSS strategy [24,30]; hence, statistical positioning results of the first four strategies are presented in this section. The relative positioning was implemented in the kinematic mode for all baseline campaigns and reset at midnight, and the final RMS was calculated for the entire 30 days. The positioning errors in time series of baseline SUT obtained by four strategies over 3 days are shown as an example in Figure 6.

**Figure 7:**
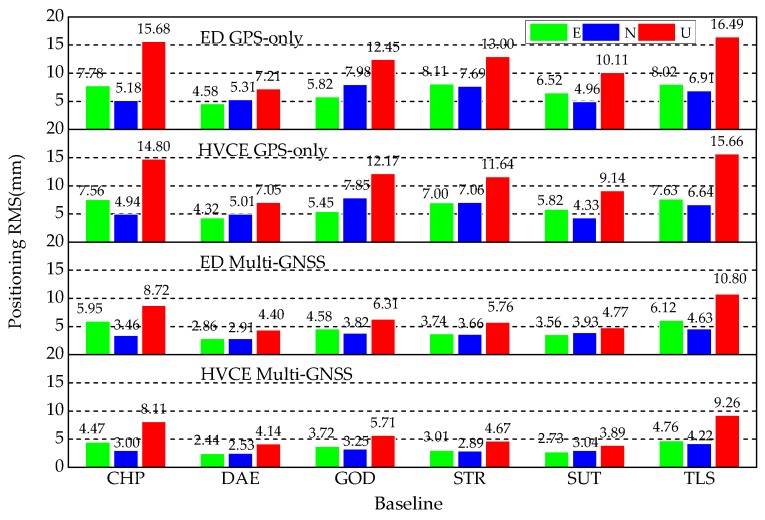
RMSs of kinematic relative positioning using four weighting strategies, from top to bottom: ED GPS-only (**first**), HVCE GPS-only (**second**), ED Multi-GNSS (**third**) and HVCE Multi-GNSS (**last**) from DoY 171 to 200 in 2019.

As mentioned in the introduction, Multi-GNSS can be beneficial to positioning accuracy compared with single-GNSS [3,4,5,6]. The RMSs of the four strategies with respect to each baseline over 30 days are shown in Figure 7, and it is obvious that the RMSs obtained by Multi-GNSS strategies are lower than by GPS-only strategies in all baselines. Table 5 presents the accuracy improvement percentages obtained by ED and HVCE Multi-GNSS strategies compared with the corresponding GPS-only strategies for all baselines. The improvement obtained by the Multi-GNSS strategy differs between baselines, but is more than 20% in every baseline. Generally, the Multi-GNSS achieves more than 30% improvement in each component in the ED method, and more than 40% in the HVCE method, compared with the corresponding GPS-only strategy.

Since the HVCE method was applied in both GPS-only and Multi-GNSS positioning, positioning improvements obtained by HVCE method were calculated, compared with the corresponding ED methods; see Table 6. In GPS-only strategy, HVCE method improves the positioning ENU accuracy by 7.4%, 6.1% and 5.9%. Meanwhile, HVCE improves the positioning ENU accuracy of Multi-GNSS strategy over GPS-only, with the improvements of 20.5%, 15.6% and 12.3%.

### 4.3. Frozen Variance of Unit Weight-Based Multi-GNSS Positioning

The previous section showed that the HVCE method improved the accuracy of Multi-GNSS positioning. However, the implementation of the HVCE method requires a heavy computational load [23]. We have calculated the computational costs of the aforementioned different positioning strategies, averaging the time consumption at adjustment process per epoch in Table 7. It is clear that GPS-only is the most time-saving strategy, because it only uses observations of common satellites one time. In contrast, the HVCE Multi-GNSS presents the highest computational cost, more than eight times that of the ED Multi-GNSS method.

**Figure 8:**
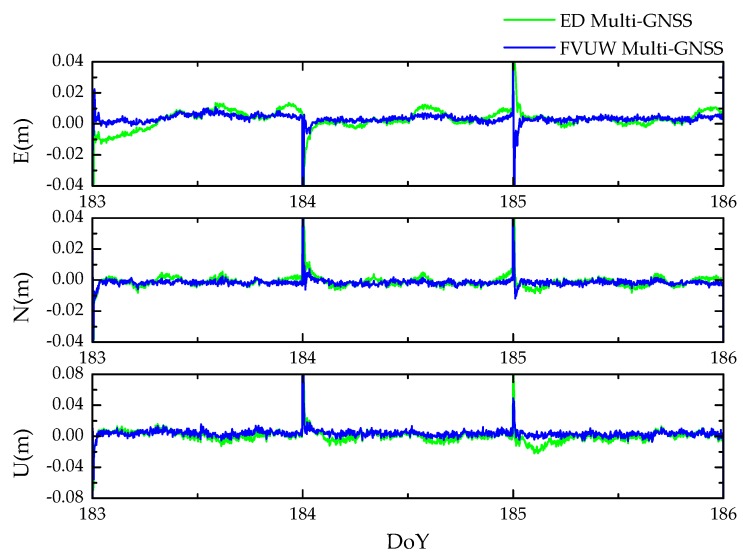
Time series of Multi-GNSS kinematic positioning errors of east (**top**), north (**middle**) and up (**bottom**) components for baseline SUT from DoY 183 to 186 in 2019. The reset appearing at the start of each day is caused by the independent daily process.

To reduce the time consumption of the HVCE Multi-GNSS method, the previously obtained variances of unit weight over 30 days in Table 4 were averaged and then frozen as a priori information in the positioning experiment, the aforementioned FVUW strategy. As no additional variances of unit weight needed to be estimated, the time consumption of the FVUW Multi-GNSS method was compared to the ED Multi-GNSS method; see Table 7. Indeed, compared to the HVCE Multi-GNSS method, the FVUW Multi-GNSS method saves 88% time on the adjustment process. The positioning performance of the FWUV Multi-GNSS method is presented in the following, and time series of positioning errors for SUT are depicted in Figure 8 as examples.

**Figure 9:**
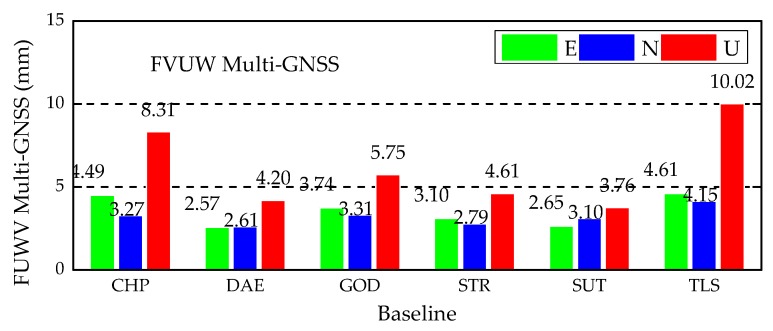
RMSs of kinematic relative positioning using FVUW Multi-GNSS positioning from DoY 171 to 200 in 2019.

The Multi-GNSS positioning RMSs of FVUW Multi-GNSS from DoY 171 to 200 in 2019 are shown in Figure 9, and are lower than the RMSs of ED Multi-GNSS shown in the third panel of Figure 7. Moreover, Table 8 shows that the Multi-GNSS positioning accuracy is improved by FVUW method with regard to ED method. Referring to Table 8, the improvement obtained by FVUW Multi-GNSS is comparable to the HVCE Multi-GNSS, as the differences between the improvement percentages of the two strategies are lower than 3% for the most of baselines. The average improved percentages of all baselines are presented in the right-most column of Table 8. Generally, the improvement obtained by FVUW method is 1% lower than HVCE, but it still improves the HVCE accuracy by more than 10%, compared with ED Multi-GNSS.

**Figure 10:**
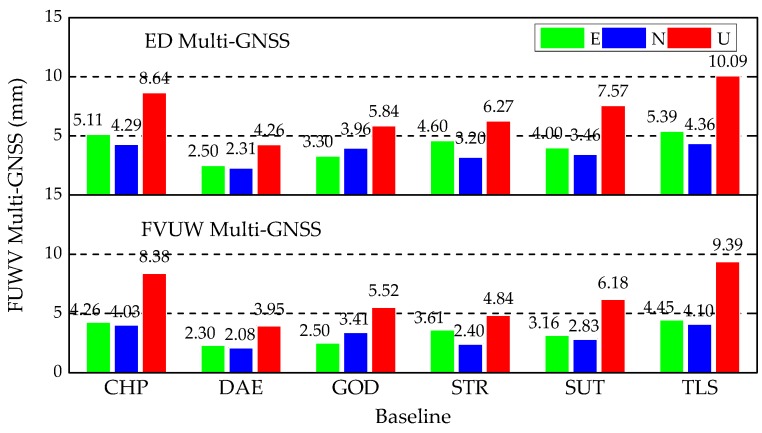
RMSs of ED Multi-GNSS (**top**) and FVUW Multi-GNSS (**bottom**) from DoY 201 to 230 in 2019.

Finally, we assessed the effectiveness of the computed frozen variances of unit weight by extending the positioning experiment for the next 30 days, from DoY 201 to 230 in 2019. The RMSs of the Multi-GNSS positioning with ED and FVUW are presented in Figure 10. The RMS values obtained by the FVUW method are lower than those obtained by the ED method in all baselines.

The average improved percentages of all baselines are presented in the right most column of Table 9, yielding comparable results to those of Table 8. At the bright side of this result, we can conclude that the positioning accuracies achieved by FVUW Multi-GNSS in the experiment extended by 30 days are as high as the improvements in the first 30 days. Specifically, the FVUW Multi-GNSS improved the positioning accuracy by more than 10% in all components, compared with ED Multi-GNSS.

## 5. Summary and Conclusions

The HVCE posterior weighting strategy has been implemented for Multi-GNSS relative positioning of six baselines formed by twelve IGS stations located at different continents. First, the basic theory of HVCE for robust Kalman filtering and the corresponding flow chart have been introduced, first to describe the algorithm implementation. Then, we evaluated its effectiveness by comparing the positioning with five strategies; namely, ED GPS-only, HVCE GPS-only, ED Multi-GNSS, HVCE Multi-GNSS and FVUW Multi-GNSS. The weight proportions of phase and code have been analyzed in detail using the averaged variances of unit weight calculated by the HVCE method. Finally, to reduce the time consumption of the HVCE method while obtaining the high accuracy positioning, the averaged variances of unit weight have been frozen as a priori information in the Multi-GNSS positioning, termed FVUW Multi-GNSS. According to the analysis and results presented in the research, the following conclusions can be drawn:

1. Multi-GNSS observations and the HVCE method improve the positioning accuracy. Compared with the corresponding GPS-only strategies, the positioning ENU accuracy is improved 34.3%, 39.5% and 45.9% by ED Multi-GNSS, and 47.9% 49.0% and 52.4% by HVCE Multi-GNSS. With respect to ED method, the HVCE method improves positioning ENU accuracy by 7.4%, 6.4% and 5.9% in the GPS-only strategy, and 20.5%, 15.6% and 12.3% in the Multi-GNSS strategy.
2. The quality of phase observations is almost equivalent among GPS, BDS, GLONASS and Galileo, as their variances of unit weight are all close to 1.0. In contrast, the quality of the code observations of different GNSS constellations differs to a great extent, presenting an average relationship as $\sigma_{R,C}^{2}>\sigma_{C,C}^{2}>\sigma_{G,C}^{2}>\sigma_{E,C}^{2}$. The $\sigma_{E,C}^{2}$ is the lowest in all baselines, which strongly indicates that Galileo has the best quality of code observations.
3. The variances of unit weights of both phase and code were quite consistent in each baseline during the 30 experimental days, which allowed the freezing.
4. Comparing with ED Multi-GNSS, the FVUW Multi-GNSS improves the positioning accuracy by 20.0%, 14.1% and 11.1% in ENU, similar to the corresponding improvements of 20.5%, 15.6% and 12.3% obtained by HVCE method. At the same time, the FVUW method saves 88% time consumption compared to the HVCE method.
5. When the frozen variances of unit weight are extended to the positioning experiment for the next 30 days, the positioning accuracy can still be improved by 18.1%, 13.2% and 10.6% in ENU, indicating the effectiveness of the frozen variances of unit weight.

In conclusion, the HVCE posterior weighting is an efficient and useful strategy for the Multi-GNSS positioning. To obtain high accuracy positioning and to reduce the time consumption of the HVCE method at the same time, we recommended using the a priori variance of unit weight from self-established experiments.

## Figures and Tables

**Table 1:** Station information and baseline distribution.

Baseline	Length(m)	Station	Receiver Type	Antenna Type
CHP	1850	CHPG	TRIMBLE NETR9	TRM59800.00 NONE
		CHPI	SEPT POLARX5	TPSCR.G3 NONE
DAE	0	DAEJ	TRIMBLE NETR9	TRM59800.00 SCIS
		DAE2	TRIMBLE NETR9	TRM59800.00 SCIS
GOD	65	GODE	SEPT POLARX5TR	AOAD/M_T JPLA
		GODN	JAVAD TRE_3 DELTA	TPSCR.G3 SCIS
STR	70	STR1	SEPT POLARX5	ASH701945C_M NONE
		STR2	TRIMBLE NETR9	LEIAR25.R3 NONE
SUT	142	SUTH	SEPT POLARX5	ASH701945G_M NONE
		SUTM	JAVAD TRE_3	JAVRINGANT_G5T NONE
TLS	1265	TLSE	TRIMBLE NETR9	TRM59800.00 NONE
		TLSG	SEPT POLARX5TR	TRM59800.00 NONE

**Table 2:** Averaged convergence time and number of available positions per day for five strategies.

ED GPS-Only	HVCE GPS-Only	ED Multi-GNSS	HVCE Multi-GNSS	FVUW Multi-GNSS
**Convergence Time (minutes)**				
18.4	16.6	6.1	5.1	5.2
**Number of Available Positions Per Day**				
459.8	462.1	475.8	477.1	476.8

**Table 3:** Average common satellite number and positioning dilution of precision (PDOP) values of different baselines.

Baseline	G	C	R	E	G + C + R + E
***Average Available Satellites Number***					
CHP	7.87	4.18	5.66	5.86	22.92
DAE	7.73	11.42	5.82	4.42	29.31
GOD	7.56	5.26	6.04	5.23	24.01
STR	7.76	10.95	5.92	5.27	29.87
SUT	7.67	7.74	5.69	5.79	26.85
TLS	7.66	5.13	6.13	5.68	24.51
***Average PDOP Value***					
CHP	1.17	2.85	1.62	1.58	0.67
DAE	1.21	1.08	1.52	2.47	0.60
GOD	1.25	2.04	1.49	1.82	0.66
STR	1.21	1.11	1.48	1.78	0.59
SUT	1.22	1.25	1.62	1.62	0.62
TLS	1.25	2.49	1.45	1.64	0.66

**Table 4:** Average phase and code variances of unit weight calculated by HVCE Multi-GNSS strategy and their corresponding root mean square (RMS) values from DoY 171 to 200 in 2019.

Baseline	$\mathrm{Phase}\;\sigma_{L}^{2}\pm RMS_{L}$				$\mathrm{Code}\;\sigma_{C}^{2}\pm RMS_{C}$			
	G	C	R	E	G	C	R	E
CHP	1.00	1.47 ± 0.57	1.47 ± 0.61	1.83 ± 0.74	3.18 ± 1.30	2.02 ± 0.87	2.68 ± 0.91	1.13 ± 0.42
DAE	1.00	2.20 ± 0.77	2.11 ± 0.92	1.53 ± 0.51	4.06 ± 1.55	4.65 ± 1.74	4.51 ± 1.61	1.33 ± 0.60
GOD	1.00	1.44 ± 0.56	1.51 ± 0.64	1.50 ± 0.51	2.66 ± 0.62	5.68 ± 2.37	8.10 ± 2.59	1.92 ± 0.76
STR	1.00	1.20 ± 0.51	1.38 ± 0.61	1.30 ± 0.54	8.28 ± 2.81	10.30 + 4.28	10.86 + 4.40	2.55 ± 0.93
SUT	1.00	1.36 ± 0.40	1.36 ± 0.47	0.88 ± 0.43	6.55 ± 3.29	12.05 ± 4.08	17.77 ± 3.75	1.61 ± 0.79
TLS	1.00	1.60 ± 0.54	1.67 ± 0.51	1.57 ± 0.67	3.34 ± 1.23	2.47 ± 1.29	3.61 ± 1.61	1.02 ± 0.40
Average	1.00	1.55 ± 0.32	1.58 ± 0.26	1.44 ± 0.29	4.68 ± 2.04	6.20 ± 3.77	7.92 ± 5.22	1.59 ± 0.52

**Table 5:** Accuracy improvement percentages of Multi-GNSS strategies based on ED and HVCE compared with the corresponding GPS-only strategies from DoY 171 to 200 in 2019.

Baseline	ED Method			HVCE Method		
	E	N	U	E	N	U
CHP	23.5%	33.2%	44.4%	40.9%	39.3%	45.2%
DAE	37.7%	45.2%	39.0%	43.6%	49.5%	41.3%
GOD	21.4%	52.1%	49.3%	31.8%	58.6%	53.1%
STR	53.9%	52.4%	55.7%	57.0%	59.1%	59.9%
SUT	45.5%	20.8%	52.8%	53.1%	29.8%	57.4%
TLS	23.8%	33.0%	34.5%	37.6%	36.4%	40.9%
Average	34.3%	39.5%	45.9%	44.0%	45.4%	49.6%

**Table 6:** Accuracy improvement percentages of HVCE GPS-only and HVCE Multi-GNSS strategies compared with the corresponding ED methods from DoY 171 to 200 in 2019.

Baseline	GPS-Only			Multi-GNSS		
	E	N	U	E	N	U
CHP	2.7%	4.6%	5.6%	24.8%	13.3%	7.0%
DAE	5.7%	5.6%	2.2%	14.7%	13.1%	5.9%
GOD	6.5%	1.6%	2.2%	18.9%	14.9%	9.5%
STR	13.7%	8.2%	10.5%	19.4%	21.0%	18.9%
SUT	10.7%	12.7%	9.6%	23.2%	22.6%	18.4%
TLS	5.0%	3.9%	5.0%	22.2%	8.9%	14.3%
Average	7.4%	6.1%	5.9%	20.5%	15.6%	12.3%

**Table 7:** Averaged time consumption at adjustment process per epoch for five strategies in the unit of millisecond.

ED GPS-Only	HVCE GPS-Only	ED Multi-GNSS	HVCE Multi-GNSS	FVUW Multi-GNSS
1	2	5	41	5

**Table 8:** Accuracy improvement percentages of FVUW method compared with ED prior weighting strategy in Multi-GNSS positioning from DoY 171 to 200 in 2019.

Components	CHP	DAE	GOD	STR	SUT	TLS	Average
East	24.4%	10.2%	18.3%	17.0%	25.5%	24.6%	20.0%
North	5.5%	10.3%	13.4%	23.8%	21.1%	10.4%	14.1%
Up	4.7%	4.5%	8.9%	20.0%	21.2%	7.2%	11.1%

**Table 9:** Accuracy improvement percentages of frozen variances of the unit weight strategy compared with the ED prior weighting strategy in Multi-GNSS positioning from DoY 201 to 230 in 2019.

Components	CHP	DAE	GOD	STR	SUT	TLS	Average
East	16.8%	8.1%	24.3%	21.5%	20.8%	17.4%	18.1%
North	6.1%	10.0%	13.9%	25.0%	18.2%	6.0%	13.2%
Up	3.0%	7.3%	5.5%	22.8%	18.4%	6.9%	10.6%
